# Heritability Estimation and Environmental Risk Assessment for Type 2 Diabetes Mellitus in a Rural Region in Henan, China: Family-Based and Case-Control Studies

**DOI:** 10.3389/fpubh.2021.690889

**Published:** 2021-07-08

**Authors:** Yinhua Feng, Xing Li, Zhenxing Mao, Wenqian Huo, Jian Hou, Chongjian Wang, Wenjie Li, Songcheng Yu

**Affiliations:** ^1^Department of Nutrition and Food Hygiene, College of Public Health, Zhengzhou University, Zhengzhou, China; ^2^Department of Epidemiology and Biostatistics, College of Public Health, Zhengzhou University, Zhengzhou, China

**Keywords:** type 2 diabetes mellitus, heritability and environment, family-based study, rural population, risk assessment

## Abstract

**Objective:** The prevalence of type 2 diabetes mellitus (T2DM) varies greatly in different regions and populations. This study aims to assess the heritability and environmental risk factors of T2DM among rural Chinese adults.

**Methods:** Thousand five hundred thirty three participants from 499 extended families, which included 24 nuclear families, were recruited in the family-based study to assess the heritable risk of T2DM. Heritability of T2DM was estimated by the Falconer method. Using conditional logistic regression model, couple case-control study involving 127 couples were applied to assess the environmental risk factors of T2DM.

**Results:** Compared with the Henan Rural Cohort, T2DM was significantly clustered in the nuclear families (OR: 8.389, 95% CI: 5.537–12.711, *P* < 0.001) and heritability was 0.74. No association between the heredity of T2DM and sex was observed between the extended families and the Henan Rural Cohort. Besides, results from the couple case-control study showed that physical activity (OR: 0.482, 95% CI: 0.261–0.893, *P* = 0.020) and fat intake (OR: 3.036, 95% CI: 1.070–8.610, *P* = 0.037) was associated with T2DM, and the proportion of offspring engaged in medium and high physical activity was higher than that of mothers in mother-offspring pairs.

**Conclusion:** People with a family history of T2DM may have a higher risk of developing T2DM, however, there was no difference in genetic risk between males and females. Adherence to active physical activity and low fat intake can reduce the risk of T2DM.

**Graphical Abstract:**
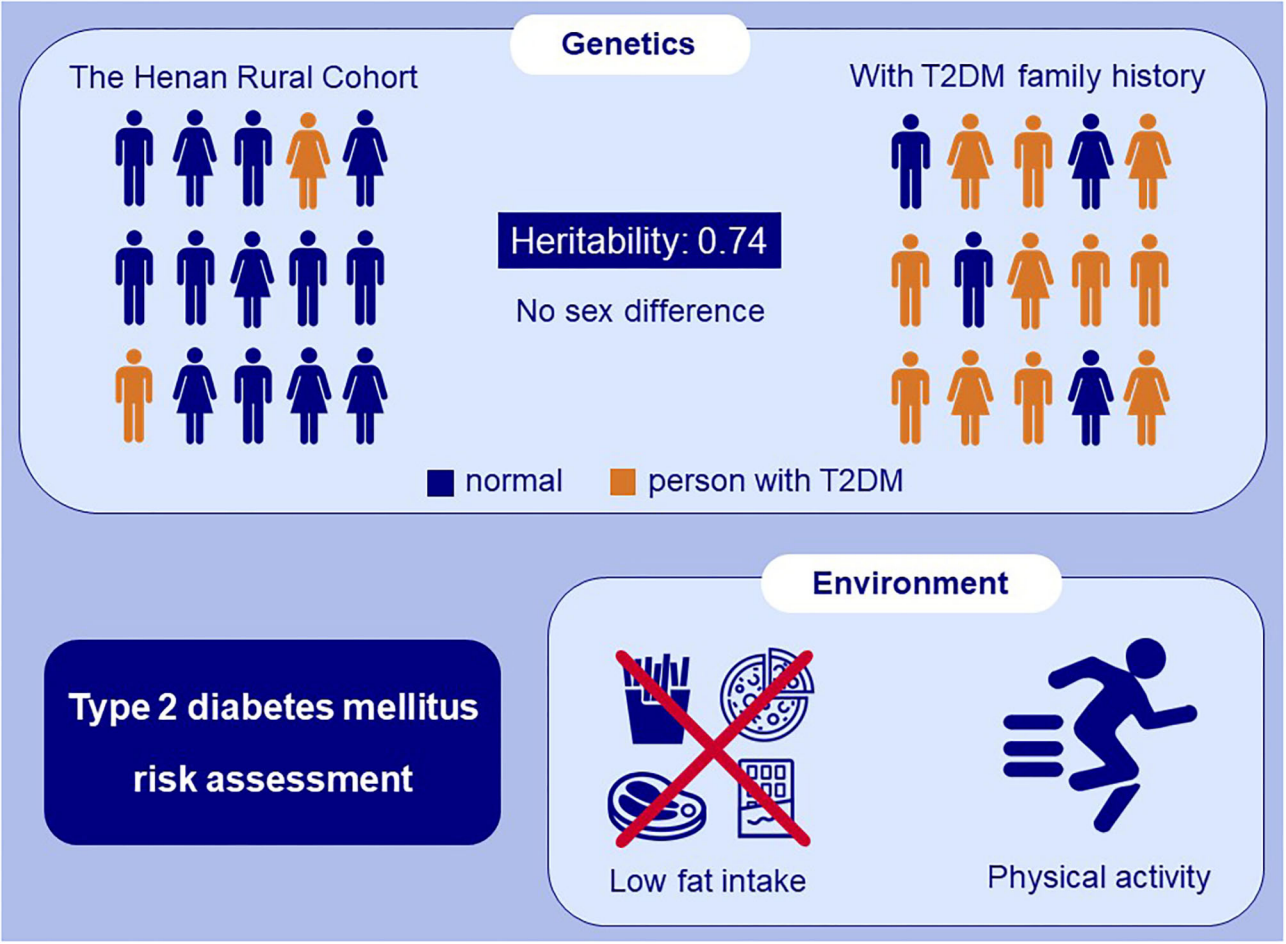


## Introduction

The prevalence of type 2 diabetes mellitus (T2DM) has increased rapidly in the past decades, causing a heavy burden worldwide. The global prevalence of diabetes among adults increased from 2.8 to 8.4% from 2000 to 2017 (1), and will reach 9.9% in 2,045 (2), according to estimates by the World Health Organization (WHO) and the International Diabetes Federation (IDF). Unlike used to be prevalent only in developed or industrial countries, T2DM is more prevalent in low-and middle-income countries currently, including China (3). It was reported that the prevalence of diabetes among Chinese adults was only 1% in 2000 and 10.9% in 2013 (4). China has become the country with the largest number of people with diabetes in the world (5).

Heritability (h^2^) refers to the degree to which genetic factors play a role in the development of disease. Large population-based studies have shown that the heritability of T2DM was estimated at 20–80% (6–8). However, there are great differences in heritability estimates among different regions and populations. For example, a family-based study in the United States showed that the heritability of plasma glucose level was 10–42% (9). However, a Finnish study showed that the heritability of T2DM was 69%, among people aged 35 to 60 years (10). The potential explanations may be the significant differences in ethnicities, regions and age ranges. In addition, previous studies have focused on developed countries, while genetic evidence for T2DM in low-and middle-income regions is still limited.

Although T2DM is strongly regulated by genetics, genetic variation does not immediately cause an epidemic of the disease within two or three generations. Therefore, the rapid increase in the prevalence of T2DM in recent decades may be mainly attributed to the environmental factors. Due to the rapid economic development, mechanization and urbanization in low-and middle-income regions, great changes have taken place in people's lifestyles (11). Excessive energy intake and sedentary have given rise to obesity which are the main risk factors for T2DM. Many classic clinical trials have shown lifestyle intervention can effectively reduce the prevalence of T2DM by controlling diet and physical activity (12–14). Therefore, as modifiable factors, environmental risk assessment in low-and middle-income areas, such as the Chinese rural areas, will greatly promote the prevention and treatment of T2DM.

Currently, heritability estimation may not be applicable to low- and middle-income areas and there is also lack of evidence on environmental risk assessment. Thus, this study aimed to assess the heritability and environmental risk factors of T2DM among rural Chinese adults to provide scientific basis for the prevention and treatment of T2DM in low-and middle-income areas. In addition, the study also explored whether sex was related to the heredity of T2DM and to also explored the association between the lifestyle of parents and that of their children.

## Materials and Methods

### Study Subjects

The family-based study, including 1,533 participants from 499 extended families, was applied to assess the genetic risk of T2DM. The study was conducted in 2013. If a family included a proband and his/her parents (both father and mother), it would be defined as a nuclear family, and their offspring and siblings were also included in the nuclear family. Twenty four families were finally identified as nuclear families and were used to assess the heritability of T2DM. All the participants are from rural areas of Zhengzhou and Wuzhi City, Henan Province.

**Figure 1:**
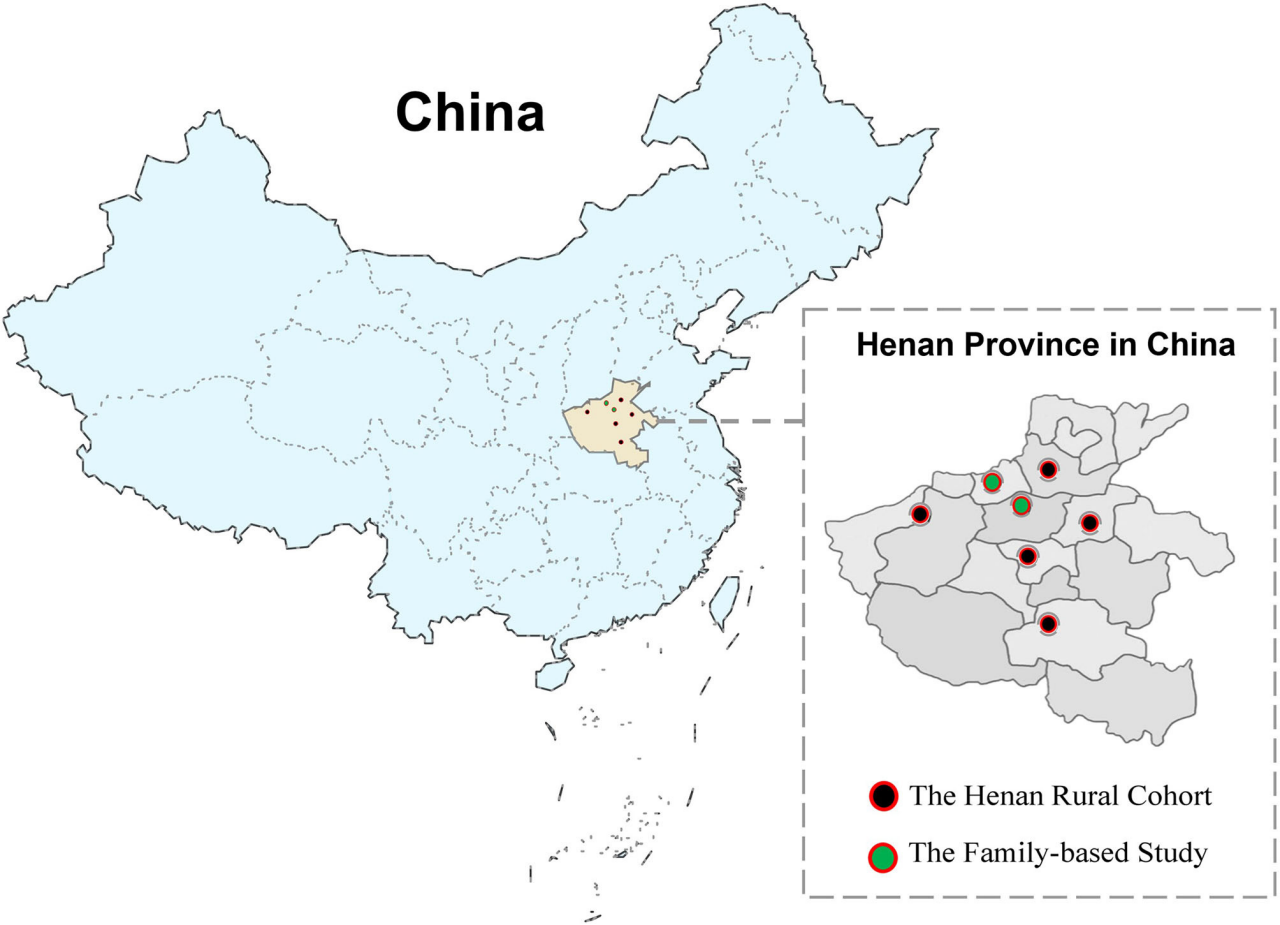
Locations of survey sites in the Henan Rural Cohort and the family-based study.

As the control of family-based study, 39,259 people were included in the Henan Rural Cohort conducted in Henan, China, whose data were collected from July 2015 to September 2017 (registration number: ChiCTR-OOC-15006699). The detailed information about the Henan Rural Cohort has been published in our previous article (15). The subjects of the family-based study and the Henan Rural Cohort Study were lived in nearby rural area (shown as Figure 1), and cluster sampling was applied in both studies.

**Figure 2:**
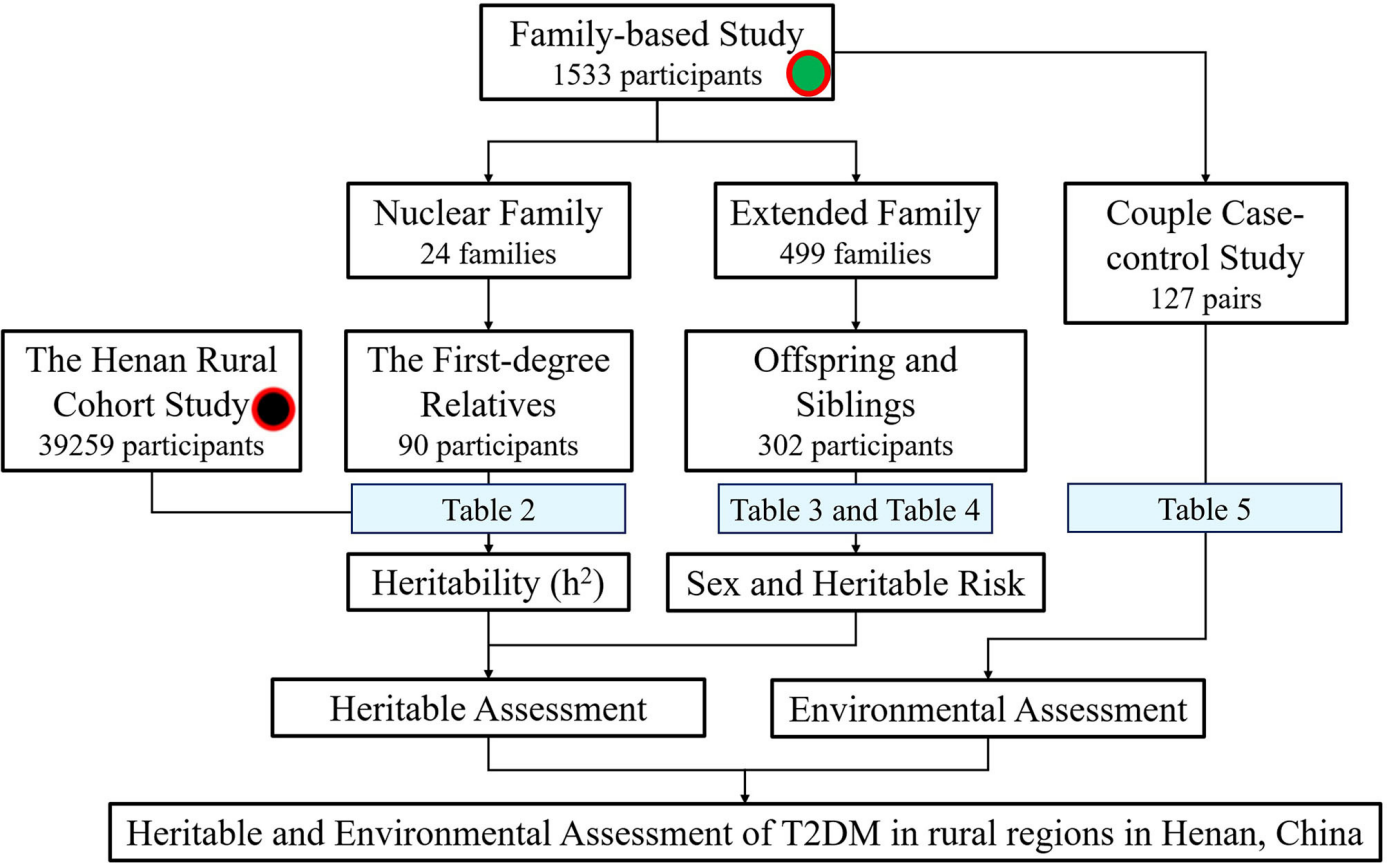
Research flowchart.

Three hundred seventy three couples were recruited, which included 229 couples without T2DM, 46 couples with their husbands having T2DM, 81 couples with their wives have T2DM, and 13 couples with both husbands and wives having T2DM. Couple case-control study, including 127 couples (*N* = 254) with only one of the spouses affected, was also used to assess the environmental risk of T2DM (shown as Figure 2).

This study complied with the Declaration of Helsinki. Zhengzhou University Life Science Ethics Review Committee [Code: (2016) MEC (S128)] reviewed and approved the protocol. All the subjects who participated in this study had provided an informed consent.

### Definition of T2DM

The diagnosis of T2DM was based on the diagnostic principles of the World Health Organization (WHO) in 1999 and the revised diagnostic criteria of the American Diabetes Federation (ADA) in 2010 (16). It was considered to be T2DM if participants had a history of T2DM, or was undergoing hypoglycemic treatment, or fasting plasma glucose (FPG) ≥ 7.0 mmol/L. Type 1 diabetes mellitus, gestational diabetes and other special types of diabetes were excluded.

### Assessment of Quantitative Traits

Data of all participants including demographic characteristic (age, sex, personal, and family history of diseases, etc.), and lifestyle (dietary habits and physical activity level), were collected using both face-to-face interviews and questionnaires.

Dietary habits included fat intake and vegetable intake, which were collected using food frequency questionnaire. The subjects were asked the frequency and intake of a certain food, which was used to calculate fat and vegetables intake. Fat intake (<50, ≥50 g/day) and vegetables intake (<50, ≥50 g/day) were classified according to the dietary guidelines for Chinese residents (17).

Using the International Physical Activity Questionnaire (IPAQ), physical activity information was collected by reviewing the intensity, frequency and time of physical activity in the past 7 days, including daily work, exercise and leisure activities. Physical activity was divided into three groups: low physical activity group, medium physical activity group and high physical activity group.

### Statistical Analysis

In this study, family-based study and couple case-control study were used to analyze the genetic and environmental risk of T2DM using the following statistical analysis methods. Chi-square test was applied to investigate the susceptibility of T2DM by comparing the prevalence of T2DM between the first-degree relatives and the Henan Rural Cohort. Chi-square test was used to analyze the relationship between sex and the heredity of T2DM. Conditional logistic regression analysis was used for assess the association between environmental risk factors and T2DM in couple case-control study, and odd ratio (OR) combined with 95% confidence interval (CI) was applied for explaining the environmental risk of T2DM. Fisher's exact test was applied to investigate the different of physical activity between probands and their parents.

Heritability is an index to measure the effect of genetic factors on disease, and it is also one of the important parameters to estimate the risk of recurrence in patients' relatives. In this study, heritability was used to estimate the role of genetic factors in the development of T2DM by using the Falconer method (18). The threshold model, proposed by Falconer in 1,965 is a simple hypothesis in the model of polygenic hereditary disease. The detailed formula is as follows:

$$\begin{array}{l} h^{2}=\frac{b}{r}\\ b=\frac{\left(X_{g}-X_{r}\right)}{a_{g}} \end{array}$$

*h*^2^: heritability; *b*: regression coefficient; *r*: coefficient of relationship (the first-degree relative of r: 1/2); *X*: normal deviate; *a*: is the mean deviation of these individuals; *q*: incidence; subscript *g*: the general population; subscript *r*: the first-degree relatives.

## Results

### Characteristics of Study Population

The characteristics of the first-degree relatives of nuclear families and The Henan Rural Cohort was shown as Table 1, which showed that there was no statistically significant difference in age, BMI, education level and marital status. In addition, 39,359 participants were included in the Henan Rural Cohort, and the prevalence of T2DM was 9.4% (males: 9.1%; females: 9.7%) (15).

**Table 1:** Characteristics of The Henan Rural Cohort Study and family-based study population.

**Variables**		**The Henan rural cohort**	**The first-degree relatives in**	***P***
		**study (*n* = 39,259)**	**nuclear families (*n* = 90)**	
Age (years)		55.6 ± 12.2	54.8 ± 18.0	0.535
Gender (*n*, %)	Male	15,490 (39.5)	46 (51.1)	0.024
	Female	23,769 (60.5)	44 (48.9)	
BMI (kg/m^2^)		24.8 ± 3.6	24.5 ± 5.9	0.431
Educational level (n, %)				0.493
	Elementary school or below	17,572 (44.7)	44 (48.9)	
	Junior high school	15,643 (39.8)	36 (40.0)	
	High school or above	6,044 (15.4)	10 (11.1)	
Marital status (*n*, %)				0.266
	Married/cohabitation	35,243 (89.8)	84 (93.3)	
	Unmarried/divorced/widowed	4,012 (10.2)	6 (6.7)	

*Continuous data are presented as mean and standard deviation [mean ± SD], and categorical data are presented as number and percentage (n, %). BMI: body mass index*.

### Estimation of T2DM Heritability

As shown in Table 2, the T2DM prevalence of the first-degree relatives of the nuclear families was significantly higher than that of the Henan Rural Cohort (OR: 8.389, 95% CI: 5.537–12.711, *P* < 0.001). In the subgroup analysis of sex, the T2DM prevalence of both males and females among the first-degree relatives was significantly higher than that among the Henan Rural Cohort (male: OR: 9.147, 95% CI: 5.116–16.354, *P* < 0.001; female: OR: 7.790, 95% CI: 4.297–14.123, *P* < 0.001). In addition, the total heritability of first-degree relatives was 0.74.

**Table 2:** The prevalence comparison of T2DM between the first-degree relatives and the Henan Rural Cohort.

	**None-T2DM (*n*, %)**	**T2DM (*n*, %)**	**OR (95% CI)**	***P***
**Total**				
Henan Rural Cohort	35,551 (99.9)	3,708 (98.9)	8.389 (5.537–12.711)	<0.001
first-degree relatives	48 (0.1)	42 (1.1)		
**Male**				
Henan Rural Cohort	14,079 (99.8)	1,411 (98.5)	9.147 (5.116–16.354)	<0.001
first-degree relatives	24 (0.2)	22 (1.5)		
**Female**				
Henan Rural Cohort	21,472 (99.9)	2,297 (99.1)	7.790 (4.297–14.123)	<0.001
first-degree relatives	24 (0.1)	20 (0.9)		

*Chi-square test was applied to investigate the susceptibility of T2DM for the first-degree relatives*.

### Association Between Sex and T2DM

No difference in the prevalence of T2DM was observed among people of different sex in the Henan Rural Cohort (*P* = 0.066) (not shown). Furthermore, the difference in prevalence of T2DM was compared between male and female offspring with paternal or maternal T2DM in extended families (Table 3). The results showed that there was no significant difference in the prevalence of T2DM among male and female offspring with paternal T2DM (*P* = 0.353). Similarly, there was also no significant difference in the prevalence of T2DM among the male and female offspring with maternal T2DM (*P* = 0.274). These results suggested that the inheritance of T2DM may not be related to sex. Furthermore, comparing the prevalence of males and females among siblings in extended families, the results showed that there was no significant association between sex and the T2DM (*P* = 0.277) (Table 4), which was also illustrated that the heredity of T2DM had no association with sex.

**Table 3:** The prevalence comparison of T2DM among male and female offspring.

**Offspring**	**None-T2DM (n, %)**	**T2DM (*n*, %)**	**OR (95% CI)**	***P***
**Father with T2DM (*****n*** **=** **71)**				
Male	39 (59.1)	2 (40.0)	2.167 (0.339–13.852)	0.353
Female	27 (40.9)	3 (60.0)		
**Mother with T2DM (*****n*** **=** **179)**				
Male	90 (54.9)	10 (66.7)	0.608 (0.199–1.858)	0.274
Female	74 (45.1)	5 (33.3)		

*Chi-square test was applied to investigate the prevalence difference between male and female offspring*.

**Table 4:** The prevalence comparison of T2DM among male and female sibling.

**Sibling**	**None-T2DM (*n*, %)**	**T2DM (*n*, %)**	**OR (95% CI)**	***P***
Male	23 (47.9)	30 (58.8)	0.644 (0.291–1.426)	0.277
Female	25 (52.1)	21 (41.2)		

*Chi-square test was applied to investigate the prevalence difference between male and female offspring*.

### Environmental Risk Assessment for T2DM

The risk of vegetable intake, fat intake, physical activity and T2DM was assessed in a couple case-control study (Table 5). Sex, age and BMI were adjusted to calculate the OR. The results suggested that individuals with high physical activity was a protective factor of T2DM (OR: 0.482, 95% CI: 0.261–0.893, *P* = 0.020). Fat intake increased the risk of T2DM (OR: 3.036, 95% CI: 1.070–8.610, *P* = 0.037). In addition, there was no statistical difference in vegetable intake between the case and the control group (*P* = 0.300).

**Table 5:** Environmental risk assessment for T2DM in couple case-control study.

**Variables**		**Control (*n* = 127)**	**Case (*n* = 127)**	**OR (95% CI)**	***P***
Vegetable intake (*n*, %)	<500 g/day	90 (70.9)	95 (74.8)	0.725 (0.394–1.333)	0.300
	≥500 g/day	37 (29.1)	32 (25.2)		
Fat intake (*n*, %)	<50 g/day	116 (91.3)	112 (88.2)	3.036 (1.070–8.610)	0.037
	≥50 g/day	11 (8.7)	15 (11.8)		
Physical activity (*n*, %)	L	39 (30.7)	60 (47.2)	Reference	
	M	30 (23.6)	26 (20.5)	0.564 (0.277–1.150)	0.115
	H	58 (45.7)	41 (32.3)	0.482 (0.261–0.893)	0.020

*Eighty one couples with T2DM wives and 46 couples with T2DM husbands were included as couple case-control study. Conditional logistic regression model was applied for risk assessment. Sex, age, and BMI were adjusted to calculate the OR. H, high physical activity; M, medium physical activity; L, low physical activity*.

### Relationship Between Physical Activity of Parents and Offspring

The differences in physical activity between probands and their parents were investigated in the family-based study (Table 6). The results suggested that in mother-offspring pairs, the proportion of offspring engaged in medium and high physical activity was higher than that of mothers (*P* = 0.010). On the contrary, there was no significant difference for physical activity between the fathers and their offspring (*P* = 1.000).

**Table 6:** The different of physical activity between probands and their parents.

		**Proband (*****n*** **=** **24)**		***P***
		**L**	**M/H**	
Father (*n* = 24)	L	8	4	1.000
	M/H	7	5	
Mother (*n* = 24)	L	12	2	0.010
	M/H	3	7	

*Fisher's exact test was applied to investigate the different of physical activity between probands and their parents. H, high physical activity; M, medium physical activity; L, low physical activity*.

## Discussion

In this study, family-based study and couple case-control study were respectively used to investigate the genetic and environmental risk factors of T2DM, respectively. According to the prevalence of T2DM in the first-degree relatives of nuclear families and the Henan Rural Cohort, the heritability of T2DM was estimated to be 0.74. In the extended families, no association between sex and T2DM was found. In addition, the results from the couple case-control study showed that high physical activity and low fat intake can reduce the risk of T2DM, and that, in mother-offspring pairs, the proportion of offspring engaged in medium and high physical activity was higher than that of mothers. Therefore, people with a family history of T2DM may have a higher risk of developing T2DM, however, there was no difference in genetic risk between males and females. Adherence to active physical activity and low fat intake can reduce the risk of T2DM (shown as Graphical Abstract).

Studies have investigated the heritability of T2DM around the world, but these results were inconsistent. A family study involving children between the ages of 8 and 19 from 184 families was conducted in Mexico to analyze the heritability of T2DM (19). This study showed that the heritability of T2DM was 50%, which was lower than our results (h^2^ = 0.74). Besides, the heritability was only 26% in previous twin study (20). One potential explanation is that older people have a higher risk of developing the disease. This was supported by the Finnish Botnia study, which showed that the heritability of T2DM in the all age groups was 25%, while the highest heritability (h^2^ = 0.69) occurred in the 35–60 years age group (10). Older people have more chances to be affected by gene-environment interaction, which may induce the development of T2DM. Another reason may be the difference in susceptibility to T2DM among ethnics. Compared with Europeans, the incidence of T2DM among Asian Indians was 2.5 times higher after adjusting for multiple confounding factors (21). In addition, the age of onset of T2DM was not the same among different ethnics. A survey of the prevalence of T2DM in 11 Asian cohorts found that the highest prevalence of T2DM was observed in the oldest age group (>70 years) in China, and among the age group 60–69 years in Singapore and India (22). Besides, the highest proportion of non-Hispanic blacks with T2DM was in their 50s, more than a decade earlier than non-Hispanic whites (23).

It is worth noting that our results suggested that there was no significant statistical difference in the prevalence of T2DM between males and females, and the heredity of T2DM may not be related to sex. Similarly, a U.S. twin cohort study showed that no sex differences were among sex found when analyzing the heritability of T2DM (24). However, the Framingham study pointed out that although there was no sex difference in the prevalence of T2DM between offspring and parents, the prevalence of T2DM in males was higher than that in females (8). The potential reasons for this difference are as follows: (1) there is no significant sex difference in the prevalence of obesity in rural China. In our previous baseline study of the Henan Rural Cohort, the prevalence of obesity was 15.9% in males and 19.0% in females, with no statistically significant difference (15). As a major risk factor for T2DM, obesity may have a similar contribution to T2DM in both males and females. (2) The risk of T2DM among older women tends to be consistent as that among men. The endocrine regulation mechanisms of women can reduce the risk of T2DM when experiencing complex metabolic changes (25, 26). However, postmenopausal women will face reduced insulin sensitivity, increased insulin resistance and a similar risk of T2DM as that of men (27, 28). It should be clear that the results on the association between sex and T2DM were not the main purpose of mostly current studies. Further prospective studies are needed to determine the relationship between sex and T2DM.

In line with previous studies, fat intake and physical activity were observed to be significantly associated with T2DM in this study, which has been confirmed by numerous studies. In the present study, the couple case-control study was used to assess the environmental risk factors, which can control the consistency of diet or exercise types to some extent, so as to better analyze the relationship between environmental factors and diabetes. With the rapid development of economy, people are commonly experiencing nutrition transitions and physical activity decline, especially in rural areas. On the basis of data from the China Health and Nutrition Survey (CHNS), the proportion of energy intake from fat among Chinese rural residents has increased from 24.6 to 58.3% in the past two decades, and fat intake has more than tripled, shifting from a high-carbohydrate diet to a high-fat diet (29). Studies from many countries have shown that nutrition transitions may be an inevitable consequence of economic prosperity (30, 31). In addition, the special dietary habits of rural residents may also be an explanation. Due to the limitations of food choice and consumption level, rural adults tend to have single type of food and higher food intake, resulting in higher fat intake and higher risk of T2DM. In addition, it has become a consensus that there was a positive correlation between high physical activity and T2DM. The China Kadoorie Biobank Collaborative Group study, included 461,211 participants, showed that physical activity substantially reduced the risk of developing T2DM (32). In rural areas, traditional agricultural activities have shifted to mechanized production, and sedentary behavior has increased (33).

Interestingly, it was observed that the proportion of offspring engaged in medium and high physical activity was higher than that of mothers in mother-offspring pairs, and there was no difference in the distribution of father-offspring pairs. A previous review summarized the association of physical activity between fathers and children, showing that 52% of the included studies had a positive correlation between physical activity of fathers and children (34). Besides, physical activity was less affected by genetic factors, which indicated that it is of great significance to increase physical activity by modifying the behavior habits of fathers, and then influence their offspring.

In this study, the participants of both family-based study and the Henan Rural Cohort Study were recruited from nearby areas, which provided more credibility to the present study. If there was a larger sample size of nuclear families, the results would have been more reliable. However, due to the late age onset of T2DM, many parents of the probands had passed away, making it difficult to increase the sample size of the nuclear families.

In conclusion, people with a family history of T2DM may have a higher risk of developing T2DM, however, there was no difference in genetic risk between males and females. Adherence to active physical activity and low fat intake can reduce the risk of T2DM. In the future, more targeted strategies are needed for the prevention of T2DM in rural areas.

## Data Availability Statement

The original contributions presented in the study are included in the article/supplementary material, further inquiries can be directed to the corresponding author/s.

## Ethics Statement

The studies involving human participants were reviewed and approved by Zhengzhou University Life Science Ethics Review Committee. The patients/participants provided their written informed consent to participate in this study.

## Author Contributions

YF drafted the original manuscript and performed the statistical analysis. XL revised the original manuscript. ZM and WH analyzed and interpreted the data and revised the original manuscript. JH reviewed the manuscript and analyzed and interpreted the data. CW and WL designed the research, reviewed the manuscript, managed and coordinated the planning, and execution of research activities. SY designed the research, managed and coordinated the planning and execution of research activities, and provided financial support for the study. All authors contributed to the article and approved the submitted version.

## Conflict of Interest

The authors declare that the research was conducted in the absence of any commercial or financial relationships that could be construed as a potential conflict of interest.
